# Transportation Arrangements for the Meeting of the Pan-American Medical Congress

**Published:** 1896-10

**Authors:** Charles A. L. Reed

**Affiliations:** Secretary International Executive Committee


### Transportation Arrangements for the Mexican Meeting of the Pan-American Medical Congress.

Dr. H. L. E. Johnson, 1400 L St., N. W. Washington, D. C., has been elected chairman of the special committee on transportation. All communications relative to rates, reservation in the special trains, etc., should be addressed to him.

A rate of one fare for the round trip has been secured between St. Louis, New Orleans, and other trans-Mississippi points, and the City of Mexico. It is confidently expected that this rate will be extended over the entire territory of the United States. Arrangements are in progress for a splendidly equipped special train of sleeping and observation cars, with first-class dining car service. Dr. Johnson will presently be in position to announce a rate, which will include railroad fare, sleeping end dining car service both ways and in the City of Mexico, and covering the expense of various side trips to the most important historic points in the republic.

CHARLES A. L. REED,
Secretary International Executive Committee.

